# Cyclodextrin-Elicited *Bryophyllum* Suspension Cultured Cells: Enhancement of the Production of Bioactive Compounds

**DOI:** 10.3390/ijms20205180

**Published:** 2019-10-18

**Authors:** Pascual García-Pérez, Sonia Losada-Barreiro, Pedro P. Gallego, Carlos Bravo-Díaz

**Affiliations:** 1 Plant Biology and Soil Science Department, Biology Faculty, University of Vigo, 36310 Vigo, Spain; pgallego@uvigo.es; 2 Physical Chemistry Department, Chemistry Faculty, University of Vigo, 36310 Vigo, Spain; cbravo@uvigo.es; 3 REQUIMTE-LAQV, Chemistry and Biochemistry Department, Science Faculty, University of Porto, 4169-007 Porto, Portugal

**Keywords:** cyclodextrins, *Bryophyllum* in vitro culture, inclusion complexes, *Kalanchoe*, antioxidants, gallates

## Abstract

The rates of production of secondary metabolites obtained by employing conventional plant breeding may be low for practical purposes. Thus, innovative approaches for increasing their rates of production are being developed. Here, we propose the use of elicited plant suspension cultured cells (PSCC) with cyclodextrins (CDs) as an alternative method for the production of bioactive compounds from *Bryophyllum* species. For this purpose, we analyzed the effects of methyl–β–cyclodextrin and 2–hydroxypropyl–β–cyclodextrin on cell culture growth and on the intra- and extracellular production of phenols and flavonoids. Results clearly show that CDs enhance the biosynthesis of polyphenols by PSCC favoring their accumulation outside the cells. CDs shift the homeostatic equilibrium by complexing extracellular phenolics, causing stress in cells that respond by increasing the production of intracellular phenolics. We also analyzed the radical scavenging activity of the culture medium extracts against 2,2–diphenyl–1–pycrilhydrazyl (DPPH) radical, which increased with respect to the control samples (no added CDs). Our results suggest that both the increase in the production of polyphenols and their radical scavenging activity are a consequence of their inclusion in the CD cavities. Overall, based on our findings, CDs can be employed as hosts for increasing the production of polyphenols from *Bryophyllum* species.

## 1. Introduction

The use of plant suspension cultured cells (PSCC) has emerged as a powerful biotechnological method for the production of plant-derived bioactive compounds of high added value that are widely employed in the cosmetic, pharmaceutical, and food industries [1]. In the last decade, the industrial applications of bioactive compounds have arisen exponentially and many commercial products obtained from PSCCs have successfully entered the market as antioxidant, anti-inflammatory, and anti-cancer ingredients, among others [1]. In practical terms, PSCC technology offers several advantages for the production of bioactive compounds with respect to the traditional ones. Remarkably, PSCC technology is totally independent of geoclimatic conditions and free of pollutants and pathogenic microorganisms, making the production of bio-antioxidants more efficient compared to conventional plant breeding techniques. Furthermore, the PSCC technology provides a rapid biomass growth and enables the production of bioactive compounds, making their scalability to industrial bioreactors easier [2], satisfying industrial requirements. Nevertheless, the use of PSCC should face several difficulties, since, in some cases, the cells may lack the biosynthetic capacity required to produce the desired compounds under the experimental conditions employed [3].

To overcome such problems, elicitation emerged as an efficient strategy to induce plant secondary metabolism in PSCCs and the subsequent increase in the biosynthesis of bioactive compounds as a result of the plant’s response to the induced stress caused by the elicitors. Elicitors are recognized by plant cells that stimulate the plant´s defense system, triggering signal transduction cascades that involve several complex intracellular signaling pathways. As a major response of plants, elicitors lead to overproduction and accumulation of secondary metabolites in plant tissue cultures [4,5].

**Scheme 1:**
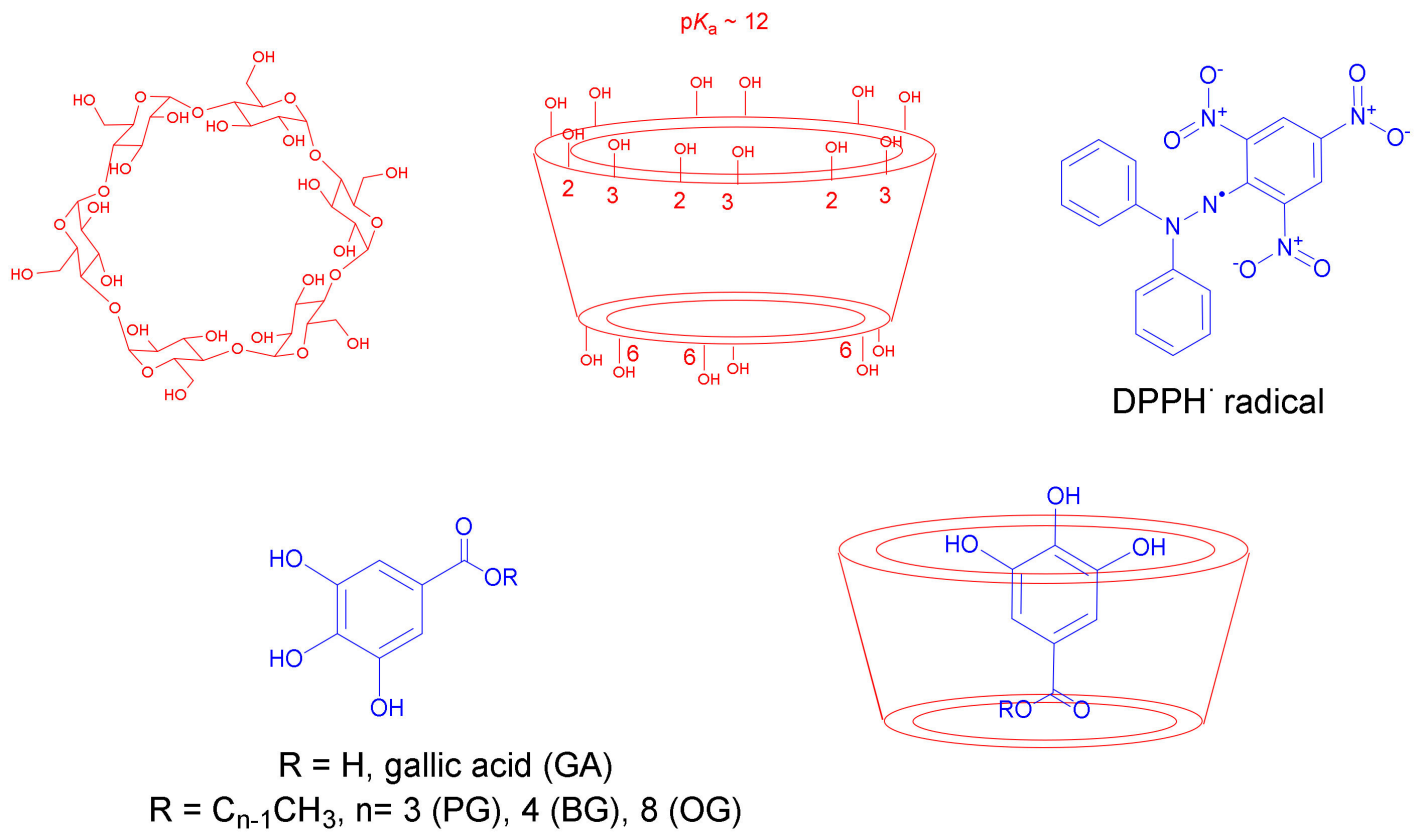
Chemical structures of β–cyclodextrin (upper row, top left and center), that of the DPPH^●^ (2,2–diphenyl–1–pycrilhydrazyl) radical employed to analyze the antioxidant activity in bulk solution (top right), and that of the antioxidants (gallic acid and derivatives) employed in this work (lower row, left). An illustrative representation of an inclusion complex between a gallic acid derivative and cyclodextrins (CDs) is that on the right of the lower row.

Specifically, cyclodextrins (CDs) have been largely used in PSCC because:

(1) CDs can be easily incorporated in PSCC, acting as “hosts” that favor the accumulation of bioactive compounds in the culture medium, from where they can be easily recovered avoiding biomass destruction [6]. Since CDs are natural compounds, the use of aqueous solutions of CDs can be considered as an ecological and environmentally friendly alternative for the extraction of potential bioactive compounds.
(2) CDs can improve the availability of hydrophobic (e.g., poorly water soluble) bioactive compounds, substantially improving their ability to cross hydrophobic cell membranes.
(3) CDs can act as efficient elicitors in PSCC due to their structural similarity (Scheme 1) to pectic oligosaccharides released from cell walls as a result of fungal infection, promoting the biosynthesis of a variety of bioactive compounds, including polyphenols [6,7], as a natural response to induced stress.

Cyclodextrins are cyclic oligosaccharides formed of glucopyranose units and can be represented as a truncated cone structure with a lipophilic cavity and a hydrophilic outer surface (Scheme 1). Natural CDs are composed of six, seven, and eight glucopyranose units and are called α–, β– and γ–cyclodextrin, respectively. The aqueous solubility of the α–, β– or γ–CDs is 145 g, 18.5 g, and 233 g dm^−3^, respectively [8], but it can be easily increased by changing the number of hydroxyl groups at the positions 2, 3, and/or 6 of the glucose moieties. For instance, the aqueous solubility of hydroxypropyl– and methyl–β–CDs is 232 and 50 g dm^−3^, respectively [8]. In general, CDs are considered safe, natural compounds, and they are widely used in parenteral nutrition and as carriers of immuno- and antitumor regulatory drugs [9]. In fact, all three natural CDs have recently been included in the European lists of additives approved for alimentary use with E–numbers E–457 (α–CD), E–459 (β–CD) and E–458 (γ–CD). Excellent reviews on their use in food and on their impact on the sensory characteristics of food, food packaging, nutrient sequestration properties, etc., are available in the literature [10,11].

The inner region of the CD cavities is hydrophobic and, if size allows, the CDs can accommodate hydrophobic compounds, forming substrate–CD inclusion complexes (e.g., antioxidant–cyclodextrin) of different stoichiometries, the most common being 1:1, 1:2, and 2:1. The substrates continuously enter and leave the CD cavities, and the formation of the inclusion complex (for clarity, a 1:1 complex) is characterized by a *K_inc_* inclusion constant defined by Equation (1). As complexes of different stoichiometry can also be formed, the equations that define the inclusion constant must be modified according to the particular stoichiometry. The formation of inclusion complexes does not imply the formation or rupture of covalent bonds, but intermolecular forces (hydrogen bonds, Van der Walls, etc.). Literature reports indicate that phenolic antioxidants (AOs), such as gallates, form 1:1 inclusion complexes with the phenolic ring inserted into the CD cavity and the –OH groups pointing outwards, Scheme 1 [8].

(1) $$AO+CD\overset{k_{F}}{\underset{k_{D}}{⇆}}AO-CD\;\;K_{inc}=\frac{k_{F}}{k_{D}}\frac{\left[AO-CD\right]}{\left[AO][CD\right]}$$

To assess the effects of CDs on bio-antioxidants production, we elicited PSCCs from *Bryophyllum × houghtonii* D. B. Ward (*Bryophyllum daigremontianum × tubiflorum*, genus *Kalanchoe*, Crassulaceae) with methyl–β–cyclodextrin (M–β–CD) and 2–hydroxypropyl–β–cyclodextrin (HP–β–CD) and determined the total phenolic and flavonoid content of both intra- and extracellular culture fractions. We also determined the radical scavenging activity of non-elicited and CD-treated PSCCs extracts using the DPPH assay, which measures the decrease of the absorbance of DPPH^●^ (2,2–diphenyl–1–pycrilhydrazyl) radical when reduced by an antioxidant (AO).

*Bryophyllum* genus includes several succulent plant species belonging to Crassulaceae family. Different *Bryophyllum* species and their derived extracts have been used in traditional medicine in Africa and Asia for the treatment of several ailments, such as diabetes and neurodegenerative and neoplastic diseases, and their beneficial health effects have been attributed to their ability to biosynthesize polyphenolic compounds such as cinnamic acid, p–hydroxybenzoic acid, vanillic acid, gallic acid, protocatechuic acid, flavonoids, bufadienolides, etc. [12].

As CDs may act as hosts of a series of AOs, we investigated the formation of inclusion complexes between gallic acid (GA) and the propyl- (PG), butyl- (BG) and octyl- (OG) derivatives with hydroxypropyl–β–cyclodextrin (HP–β–CD) and their antioxidant activity. These compounds were chosen as a representative set of AOs with different hydrophobicity because GA is a common phenolic compound that is present in a variety of plant extracts, including *Bryophyllum* species [12,13]. Therefore, the present study also aims to provide valuable knowledge on the formation of AOs inclusion complexes from plant extracts with CDs. Indeed, the use of CDs to promote the production of bioactive compounds in cell cultures may constitute a sustainable and environmentally friendly biotechnological approach with important added value for human health and the treatment of a number of diseases.

## 2. Results and Discussion

### 2.1. Kinetics of the B. × houghtonii Suspension Cultured Cells (BHSCC) Growth: Effects of Cyclodextrins (CDs)

To determine the suitability of CDs as elicitors in *B.* × *houghtonii* suspension cultured cells (BHSCC), we carried out a kinetic study to analyze their effects on cultured cell growth. Details are given in the experimental part, Section 3.5. The effects of CDs on PSCC growth are difficult to predict because discrepant or even contradictory results were reported in the literature, leaving their role in PSCC unclear. For instance, Perassolo et al. [14] and Zhou et al. [15] reported an increase in growth of PSCC from *Morinda citrifolia* and from *Catharanthus roseus*, treated with 20 mM HP–β–CD and 10 mM β–CD, respectively. Durante *et al.* [16] reported a negligible effect of CDs on cell growth when employing PSCC from *Artemisia annua* treated with 50 mM M–β–CD. In contrast, a reduction on PSCC growth from *Daucus carota* was reported when they were treated with 50 mM M–β–CD [17], as well as cell cultures from *Vitis vinifera,* under the same conditions [18].

**Figure 1:**
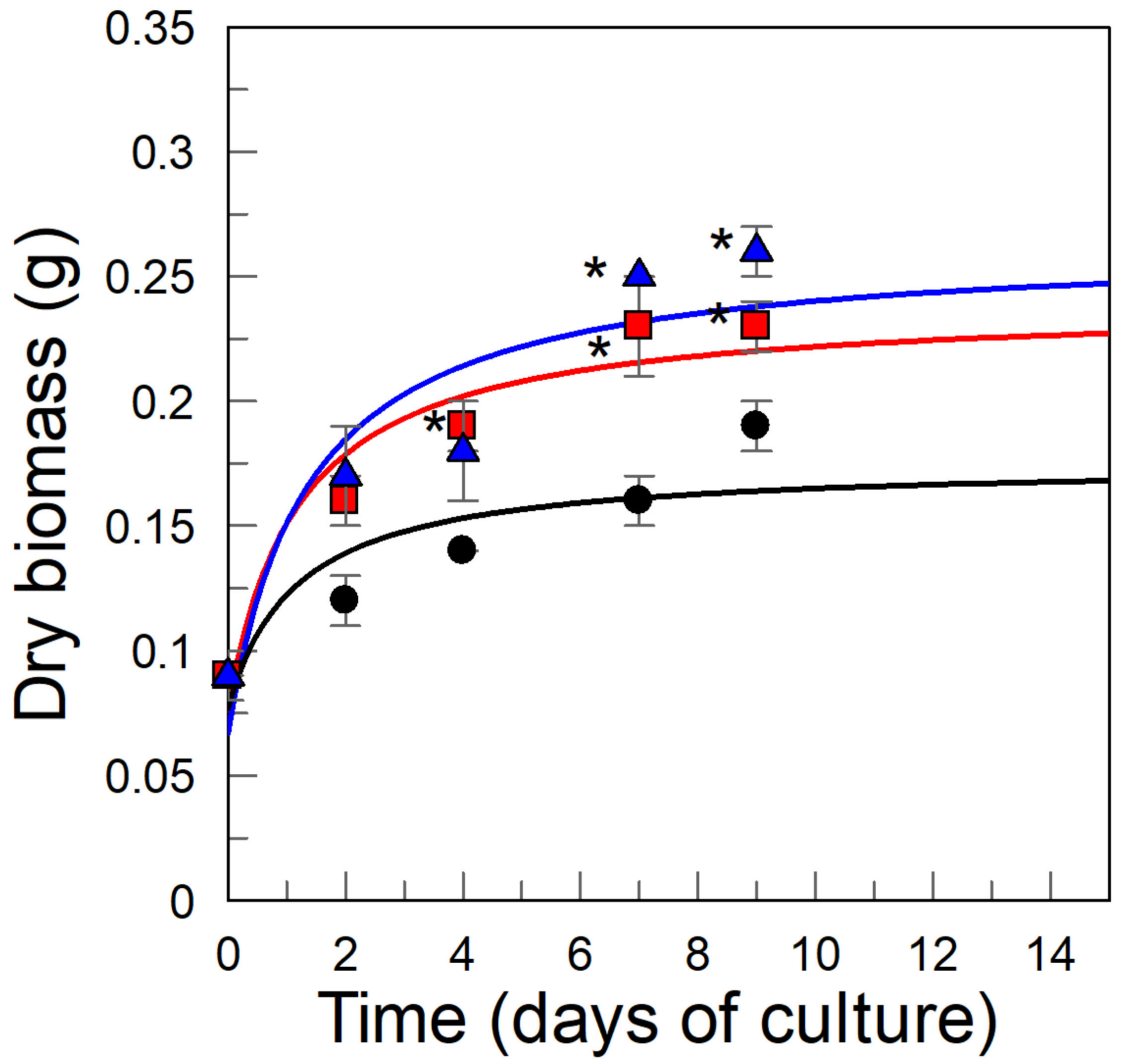
Kinetics of *B.* × *houghtonii* suspension cultured cells (BHSCC) growth, expressed as the change of the grams of dry biomass produced over time, in the absence of CDs (control, -●-) and in the presence of 50 mM M–β–CD (-■-) and HP–β–CD (-▲-). Values are given as the mean ± SD of three independent replicates, and (*) refers to significant differences between control and treatments (*p* < 0.05). Solid lines are the theoretical curves obtained by fitting the experimental data to a Michaelis-–Menten type equation.

Thus, we first determined the effects of CDs on BHSCC cell growth to analyze if CDs can be added to PSCCs without interfering with the viability of regular cell culture. Figure 1 shows the growth kinetics of BHSCC, expressed as the variation of the amount (grams) of dry biomass produced in the presence and absence of CDs over time. Either in the presence or absence of CDs, saturation kinetics were obtained.

It is worth noting that the amount of dry biomass produced was independent of the nature of the CD employed, with differences less than 11% at day 9. However, the production of dry mass increased 1.5-fold with respect to the value in the absence of CDs (0.17 ± 0.01 g), highlighting the significant effect of the presence of CDs on the amount of dry biomass produced. The average BHSCC cell growth rate (determined by employing Equation (2), Section 3.5) was 0.12, 0.17, and 0.21 day^−1^ for control and elicited plant cultures, with M–β–CD and with HP–β–CD, respectively. The kinetic profiles reveal that, in the presence of CDs, the saturation limit was reached after 9 days and, therefore, this cultivation time was set for subsequent studies on the effects of CDs. The maximum amounts of dry weight produced were 0.23 ± 0.01 g and 0.26 ± 0.01 g when plant extracts were grown in the presence of M–β–CD and HP–β–CD, respectively.

### 2.2. Intracellular and Extracellular Production of Total Phenolic Compounds in BHSCC: Effects of Added CDs 

To analyze the effects of CDs as potential elicitors, we determined the total phenolic content (TPC) in both the intracellular and extracellular fractions. For the purpose, we prepared hydromethanolic extracts of both the cellular fraction of suspension cultures and the fraction of culture medium. TPC values were expressed as milligrams of gallic acid equivalents per liter of suspension (GAE, mg/L). Details of the procedure are given in the experimental Section 3.6 and Section 3.7.

**Scheme 2:**
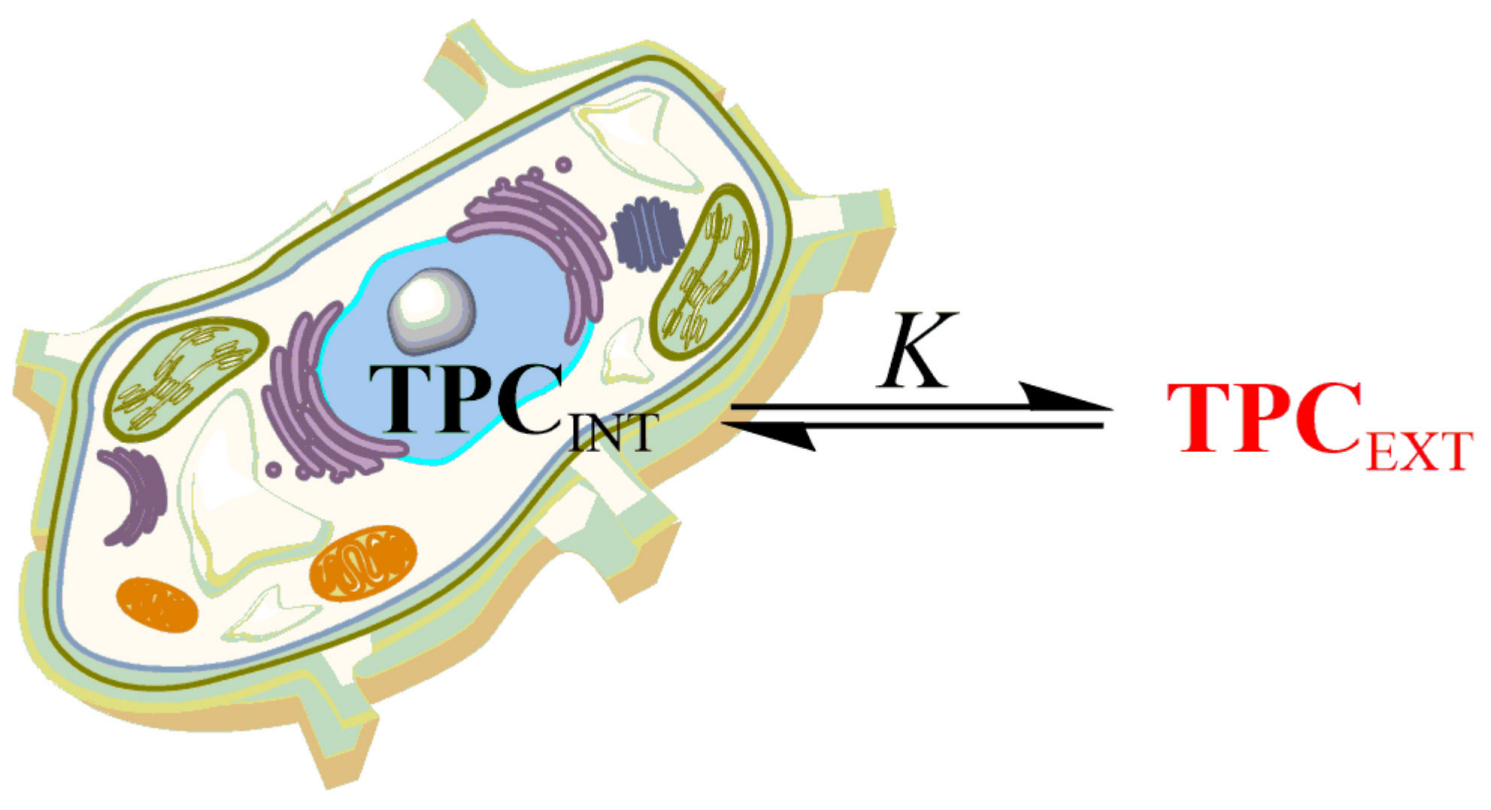
Illustrative representation of the homeostatic equilibrium between the intra- and extracellular medium of the cell produced polyphenolic compounds.

**Figure 2:**
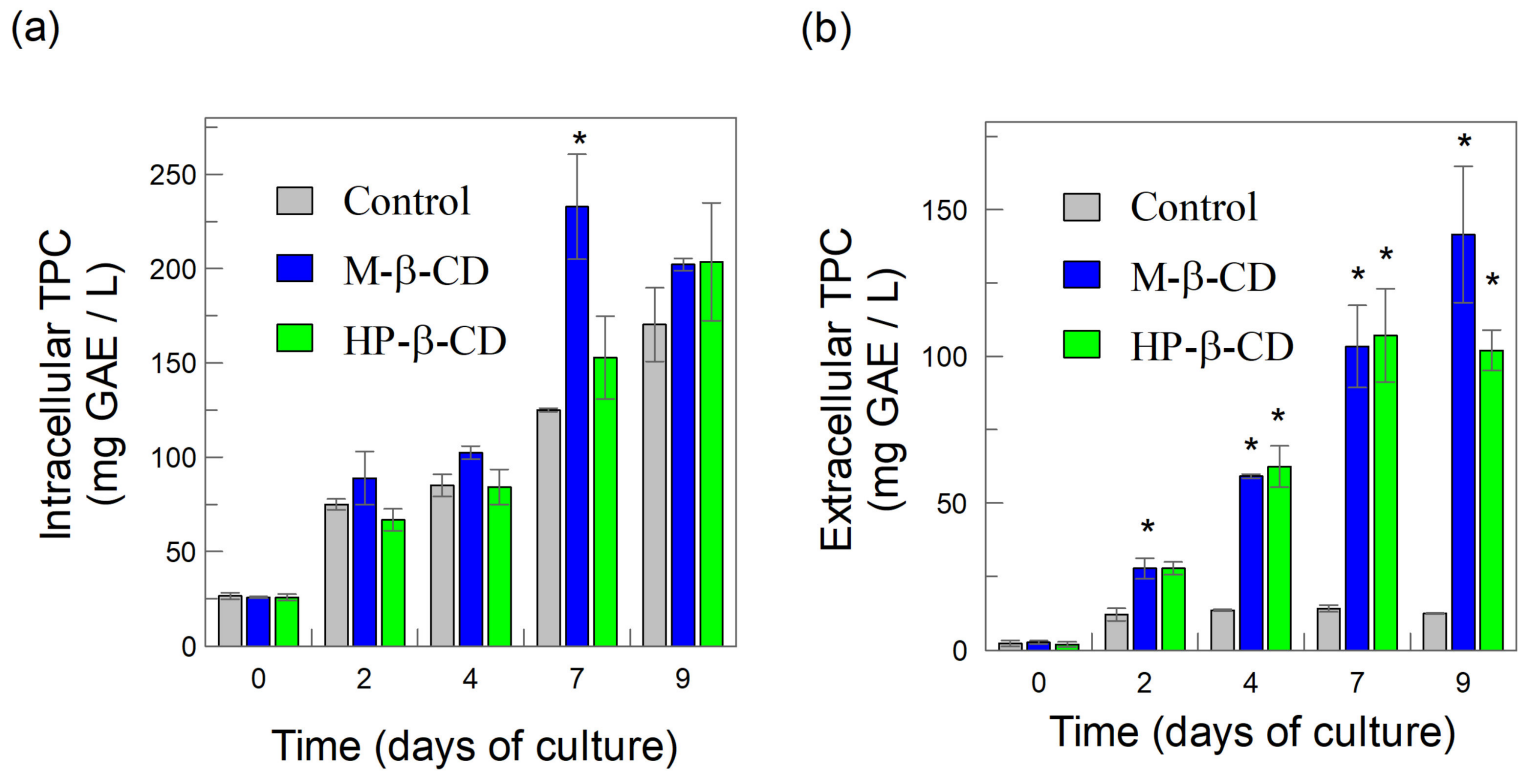
Variation of the intracellular (**a**) and extracellular (**b**) phenolic content (or production) expressed in terms of total gallic acid equivalents (GAE, mg/L) in non-elicited (control) and cyclodextrin (CD)-treated *B.* × *houghtonii* suspension cultured cells BHSCC extracts ([CD] = 50 mM) over time. Values are given as the mean ± SD of triplicates and (*) refers to significant differences between control and treatments for each day (*p* < 0.05).

Figure 2a,b show the variations of the intracellular and extracellular TPCs over time, respectively. At t = 0 and in the absence of CDs, the intracellular TPC value is much higher than the extracellular TPC value, indicating that most secondary metabolites are produced inside the cells but a portion of them partition to the culture medium so that the ratio between the extracellular and intracellular TPC is *K* ≈ 0.1, Scheme 2.

The intracellular TPC increases with time and no significant variation was observed after day 9. We also observed that, in general, the detected increase of TPC with time was essentially independent of the presence or absence of CDs with the exception of the high value of 233 ± 28 mg GAE per liter observed in the presence of M-β-CD at day 7 (Figure 2a). The high value of this peak can be attributed to various reasons which, in any case, do not change the main conclusions of the work. 

The results are in agreement with previous results showing a negligible effect of HP–β–CD (20 mM) on the intracellular TPC in PSCC from *M. citrifolia* [14] and of M–β–CD (50 mM) on the intracellular TPC in PSCC from *Linum usitatissimum* [6], respectively.

Figure 2b shows that, in the absence of CDs, the extracellular TPC barely increases during the days of culture, however, the determined values at a given time are always much lower than those of the intracellular TPC. Addition of CDs substantially increases the determined extracellular amount of TPC. For instance, at day 9, the ratio between the TPC obtained in the presence of CDs, compared to that in their absence, was ~7.9 fold (M–β–CD) and ~8 fold (HP–β–CD).

**Scheme 3:**
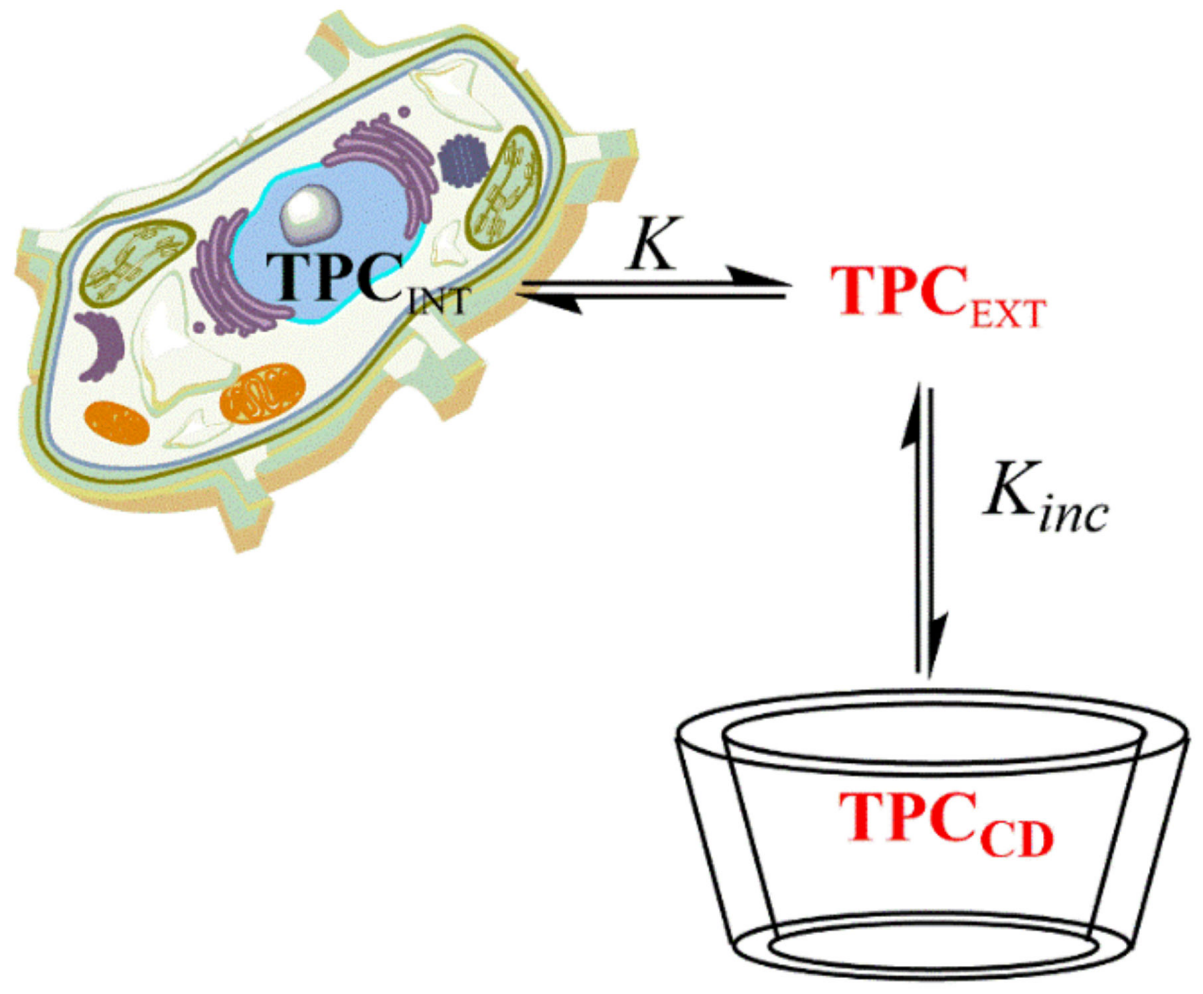
Illustrative representation of the different equilibria involved when cyclodextrins (CDs) are used as elicitors. CDs are capable of hosting hydrophobic molecules inside their cavities forming inclusion complexes with inclusion constants, *K_inc_*. The formation of such complexes reduces the amount of “free” extracellular TPC shifting, in turn, the equilibrium between the intracellular (TPC_INT_) and extracellular (TPC_EXT_) phenolic content, causing a stress to the cell so that the cell is compelled to increase the production of TPCs to maintain the homeostatic equilibrium.

Enhancement of extracellular levels of phenols in PSCC elicited with CDs has already been reported in the literature. For example, in cells elicited with M–β–CD (50 mM), vanillin concentration was boosted at the extracellular level in PSCC from *D. carota* and trans-resveratrol was exogenously accumulated in PSCC from *V. vinifera* [17,19]. The reasons for such an increase are generally attributed to the presence of elicitors, which trigger the stress response in the cell with a concomitant enhancement on the intracellular production of secondary metabolites. In this study, the amount of TPC in the extracellular medium, in the presence of CDs, was much higher than in their absence, and therefore it is clear that the presence of CDs plays an essential role in driving the overproduction of TPC. We hypothesize that it may be a consequence of the ability of CDs to host hydrophobic phenolic compounds (and other chemicals) inside their cavities, as illustrated in Scheme 3.

As extracellular phenolics are complexed, TPCext decreases. Cells are then stressed and forced to maintain their homeostatic equilibrium. Consequently, cells should increase their phenolic production in the presence of CDs, which are displaced to the culture medium. This phenomenon is, conceptually, quite similar to the Le Châtelier´s chemical principle, expressed as “If a dynamic equilibrium is disturbed by changing the conditions, the equilibrium position moves to counteract the change”.

To prove or disprove our hypothesis, we performed a series of experiments to determine the inclusion constants of several representative antioxidants. These experiments are described below in Section 2.5. However, prior to that, we carried out some experiments in order to learn more about the effects of CDs as elicitors on BHSCC. Specifically, we analyzed the effects of CDs on the production of flavonoids and the antioxidant activity of the secondary metabolites (phenols and flavonoids) elicited.

### 2.3. Cyclodextrins (CDs) as Elicitors: Effects on the Intracellular and Extracellular Production of Flavonoids from B. x houghtonii Suspension Cultured Cells (BHSCC)

**Figure 3:**
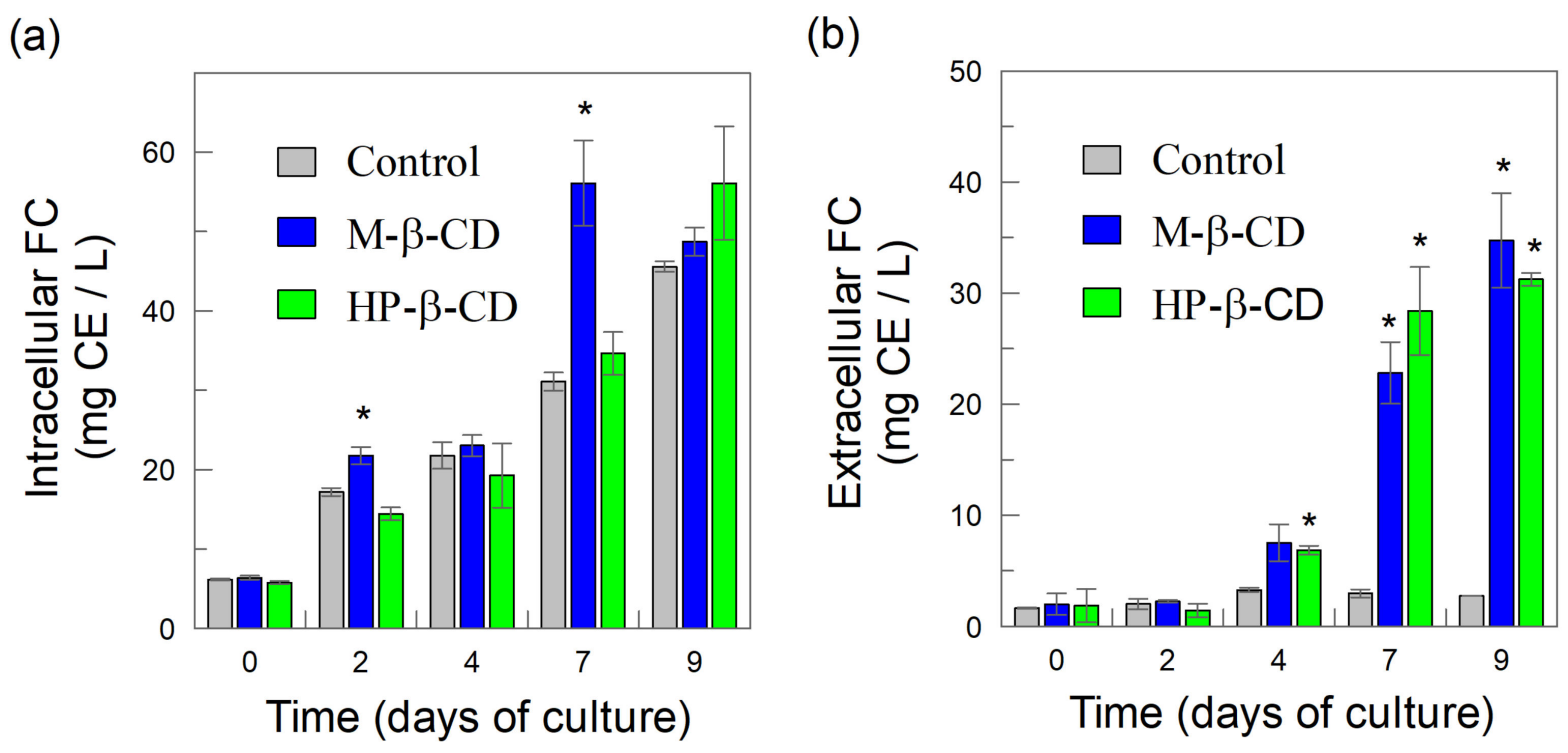
Variation of the intracellular (**a**) and extracellular (**b**) flavonoid content (or production) in non-elicited (control) and cyclodextrin (CD)-treated *B.* × *houghtonii* suspension cultured cells (BHSCC) extracts ([CD] = 50 mM) over time. FC production is expressed as total catechin equivalents (CE, mg/L). Values are given as the mean ± SD of triplicates and (*) refers to significant differences between control and treatments for each day (*p* < 0.05).

Figure 3 shows the intracellular and extracellular flavonoid content (FC), which is expressed in terms of equivalents of catechin (CE, mg/L). Details on the procedure are given in the experimental Section 3.8.

Intracellular FC increases over culture time, and the presence or absence of CDs did not have a significant effect on their production, with differences less than 16%, after 9 days of cultivation, Figure 3a. Similarly, the pattern for the intracellular flavonoid production (Figure 3a) followed the same trend to that obtained for intracellular TPC production, Figure 2a.

The extracellular FC at *t* = 0 was much lower than the intracellular FC, suggesting again that most of the flavonoids were produced inside cells and only a small fraction of them were released to the culture medium. The ratio between the extracellular and intracellular FC content was K ≈ 0.3 at that time. Additionally, Figure 3b shows that, in the absence of CDs, the extracellular FC remains essentially constant up to 9 days of cultivation. In the presence of CDs, the extracellular FC remains constant up to 2 days and then increases significantly over the culture time so that at day 9, the ratio between the FC in the presence of CDs is of ~17.3 fold (M–β–CD) and ~16.6 fold (HP–β–CD), respectively.

The delay in flavonoid production with respect to total phenolic compounds content has already been reported in the literature and is usually attributed to the additional time required by PSCC to synthetize flavonoids as a consequence of the metabolic flux across biosynthetic pathway [20]. In fact, the biosynthesis of flavonoids involves the formation of complex structures that need the accumulation of sufficient chalcone scaffolds, which subsequently undergoes the induction and action of a large group of heterogeneous biosynthetic enzymes [20]. CDs have been shown to induce key genes related to flavonoid biosynthesis, such as chalcone synthase (CHS), which is the enzyme responsible for the synthesis of the aforementioned chalcone scaffolds [21]. Hence, flavonoid biosynthesis requires a previous accumulation of simpler phenolic compounds to be synthesized. [22] The observed delay could be also attributed to the encapsulation of simpler phenolic compounds needed to bio-synthesize flavonoids. Consequently, as our findings suggest, a significant increase in TPC was observed on day 2, prior to flavonoid biosynthesis and production by BHSCC treated with CDs, that occurred at day 4 (Figure 2b and Figure 3b). In parallel, the increase in FC can be a consequence of the stress produced by CDs, which form inclusion complexes with flavonoids, decreasing their extracellular concentration in the free form. Therefore, the equilibrium shifts between the extracellular and intracellular FCs, and cells are driven to produce more flavonoids to maintain its homeostatic equilibrium, Scheme 3.

### 2.4. Radical Scavenging Activity of Intracellular and Extracellular Phenolics and Flavonoids: Effects of Cyclodextrins (CDs) 

Secondary metabolites have a wide range of industrial applications, including their use in medicine and, therefore, they serve as candidates for commercialization in pharmaceutical preparations. Thus, the potential use of the polyphenolic and flavonoid extracts as radical scavengers, to minimizing the harmful effects produced by reactive oxygen species, should be investigated [23]. To that end, we determined the radical scavenging activity (RSA) of BHSCC-derived extracts using the Brand-Williams method [24], which exploits the reaction of the antioxidants with the stable free-radical DPPH^●^, whose chemical structure is shown in Scheme 1. This method is appropriate and convenient because it can evaluate the antioxidant activity over time, since most phenolic antioxidants react slowly with DPPH^●^. However, we are aware that it has a limited relevance to biological systems [25]. RSA is usually expressed in terms of percentage of inhibition of the radical content (%Inhibition), and details of the method and calculations are given in Section 3.9.

**Figure 4:**
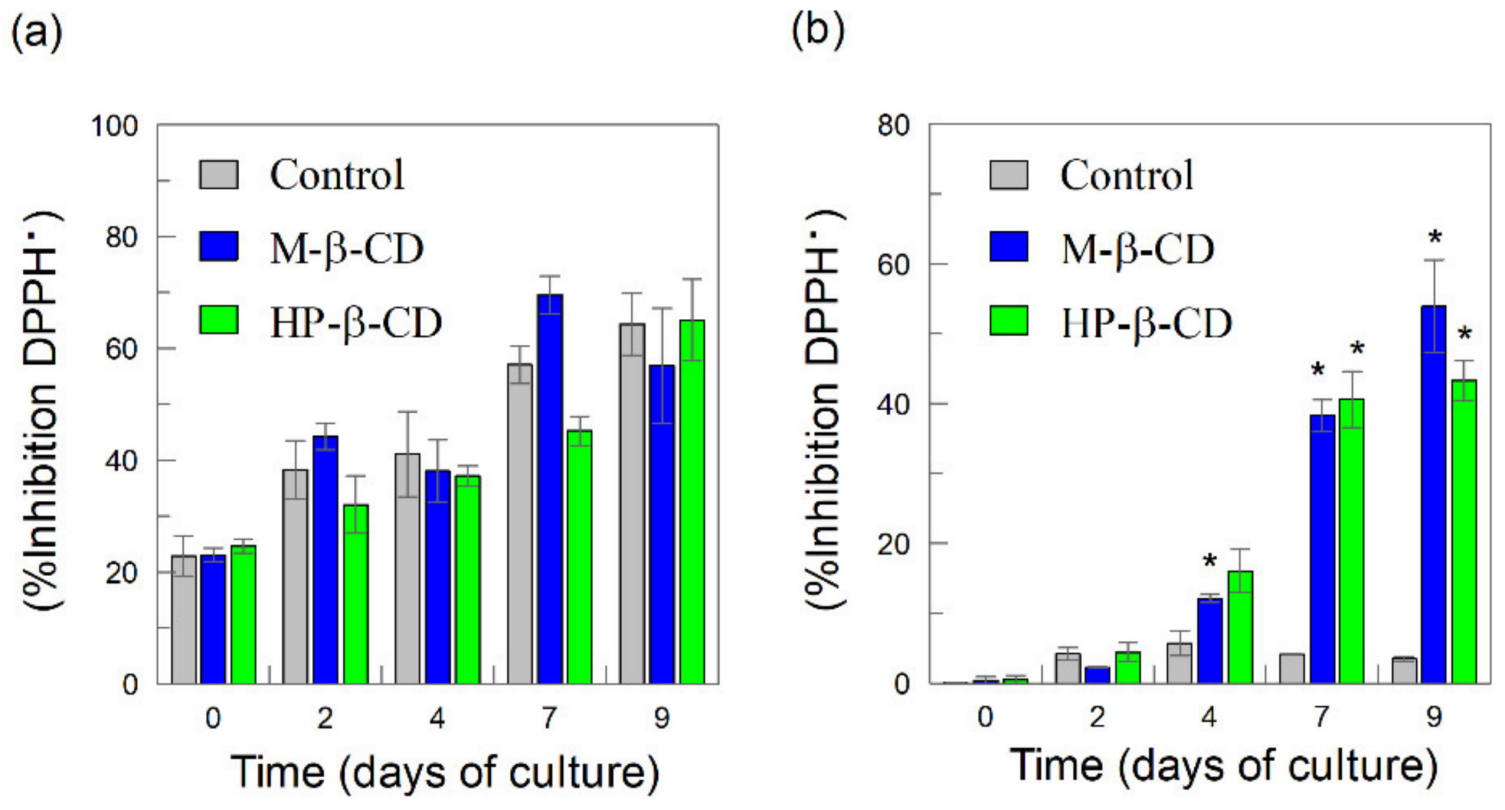
Radical scavenging activity (RSA) of intracellular (**a**) and extracellular (**b**) fractions from *B.* × *houghtonii* suspension cultured cells (BHSCC) in the absence (control) and in the presence of cyclodextrins (CDs). RSA values are expressed as %Inhibition (Equation (3), Section 3.9), [CD] = 50 mM at T = 25 ± 1 °C. Values are given as the mean ± SD of triplicates and (*) refers to significant differences between control and treatments for each day (*p* < 0.05).

Figure 4 shows the percentage of inhibition obtained from intra-and extracellular fractions for both elicited and non-elicited BHSCC extracts. Figure 4a shows the RSA for the intracellular polyphenols in the presence and absence of CDs and no significant differences (less than 12–18%) were detected, regardless of the time of culture. This is consistent with the negligible effect of CDs on the production of intracellular TPC and FC, as described previously.

**Figure 5:**
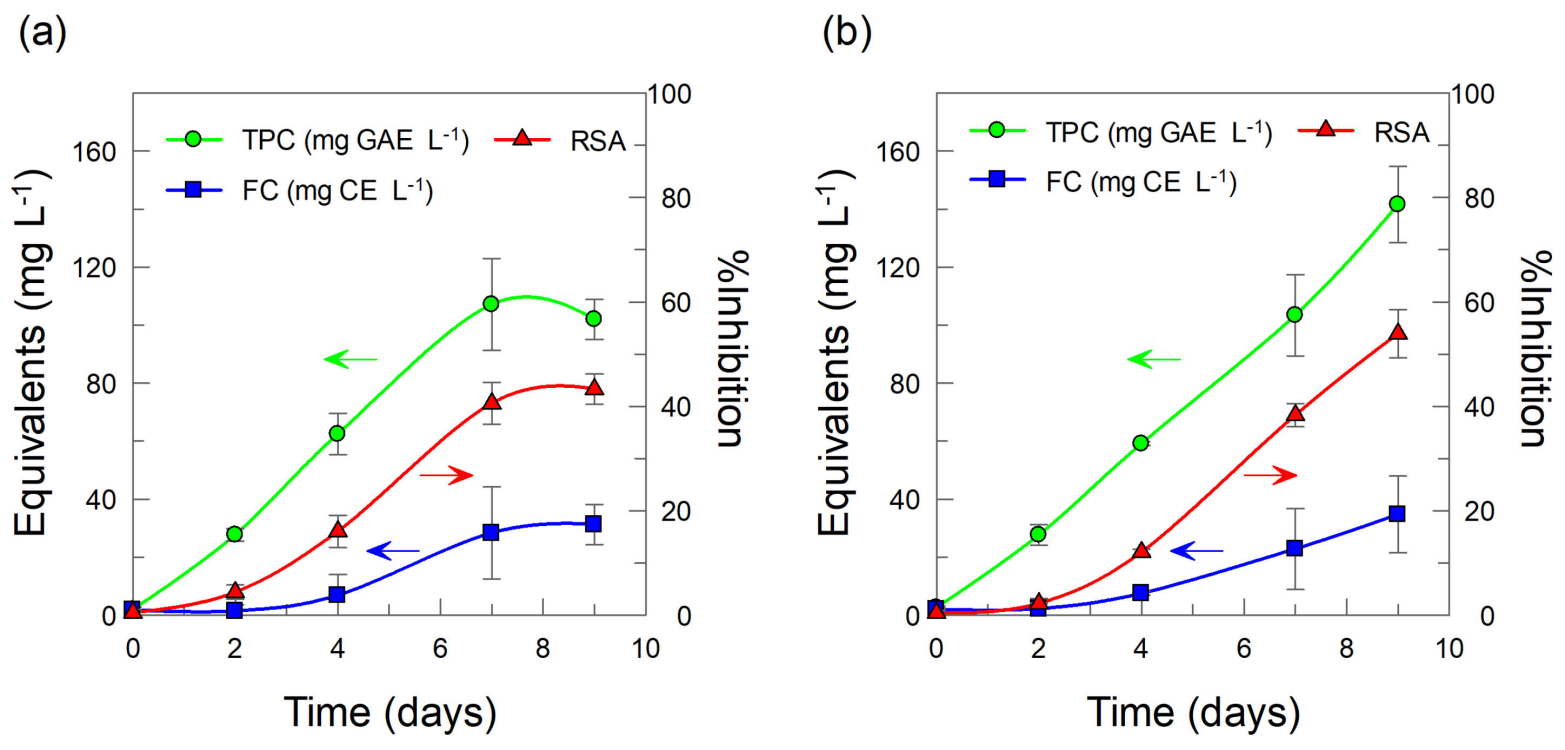
Extracellular total phenolic content (TPC, -●-) and flavonoid content (FC, -■-) and extracellular radical scavenging activity (RSA, -▲-) of extracts from *B.* × *houghtonii* suspension cultured cells (BHSCC) treated with M–β–CD (**a**) and HP–β–CD (**b**); [CD] = 50 mM at T = 25 ± 1 °C. Values are given as the mean ± SD of triplicates. The solid lines are drawn to aid the eye.

In contrast, statistically significant differences were obtained when analyzing the RSA of extracellular polyphenols in the presence and absence of CDs, Figure 4b. The RSA values obtained for the control samples were very low and constant over the time. However, in the presence of CDs, the extracellular RSA values increased significantly (*p* < 0.05) over the time. For instance, at day 9, the RSA value was 3.45 ± 0.37 for the control and, in the presence of CDs, it increased up to RSA = 54 ± 7 (M–β–CD) and 43 ± 3 (HP–β–CD). That supposes an increment in RSA values of ~12–16-fold in elicited cultures, following the same trend as observed for TPC and FC in this study. In addition, the variation on the extracellular RSA values over time shows a time–course pattern, in accordance with TPC and FC accumulation (Figure 5).

The increase in RSA values from CD-treated cultures could be attributed to the extracellular TPC and FC accumulation. This hypothesis, however, can be disputed because the homeostatic equilibrium in the cell must be maintained and, thus, the amount of “free” extracellular polyphenols and flavonoids should be constant. Nevertheless, cyclodextrins form inclusion complexes with polyphenols and flavonoids, and they may alter their radical scavenging ability as a result of the inclusion process that takes place in the culture medium, Scheme 3. Our results strongly support the hypothesis that the radical scavenging ability of polyphenols is enhanced by their encapsulation with CDs. The hypothesis is in agreement with literature reports that suggest that RSA values from complexed antioxidants are significantly higher [26,27,28]. To assess this hypothesis, we carried out a new experiment on the complexation of some representative antioxidants and investigated their radical scavenging activity in the presence of CDs.

### 2.5. Determination of the Inclusion Constants of Representative Polyphenols with Cyclodextrins (CDs) (K_inc_)

The inclusion constants (*K_inc_*) of a representative set of AOs derived from gallic acid and their radical scavenging activity, in the presence and absence of CDs, were determined as described in Section 3.10. The complexation equilibrium between a substrate (e.g., an antioxidant) and a CD is defined by the equilibrium constant given in Equation (1).

Table 1 displays the obtained *K_inc_* values for some gallic acid derivatives and, for comparative purposes, literature values for the inclusion constants of polyphenols commonly presented in *Bryophyllum* extracts [12,13] are also included.

In general, the determined *K_inc_* values for the formation of inclusion complexes with HP–β–CD were higher than those with the native β–CDs, most probably because of the terminal groups of the HP–β–CD, that facilitate the formation of stronger hydrogen bonds with the antioxidant [29]. Changes in the hydrophobicity of gallates have a significant effect on the *K_inc_* values, increasing ~14.5 times upon increasing the number of C atoms in the alkyl chain length of the antioxidant from 3 to 8. Results in Table 1 suggest that CDs, mainly HP–β–CD, may complex effectively polyphenolic compounds and, thus, CDs behave as effective hosts in the culture medium of BHSCC [11].

### 2.6. Effect of Cyclodextrins (CDs) on the Radical Scavenging Activity of Gallic Acid Derivatives

Cell extracts contain a mixture of a number of antioxidants, and the radical scavenging activity of the mixture may not be the sum of the RSA of individual AOs because synergistic, antagonistic, or additive effects may take place. Whatever the predominant effect, the formation of inclusion complexes with CDs may affect the RSA values of the AOs measured in the absence of CDs, and thus we analyzed the effects of β–CD on the radical scavenging activity of various alkyl gallates, such as propyl gallate (PG), butyl gallate (BG), and octyl gallate (OG). β–CD was used in the experiments as a model to show that the complexation of phenolics affects their RSA values. RSA values were evaluated by employing the stable free-radical 2,2–diphenyl–1–pycrilhydrazyl (DPPH^●^) and the scavenging activity was assessed in terms of percentage of inhibition of the radical content (%Inhibition) of the samples, as indicated in Section 3.9.

**Figure 6:**
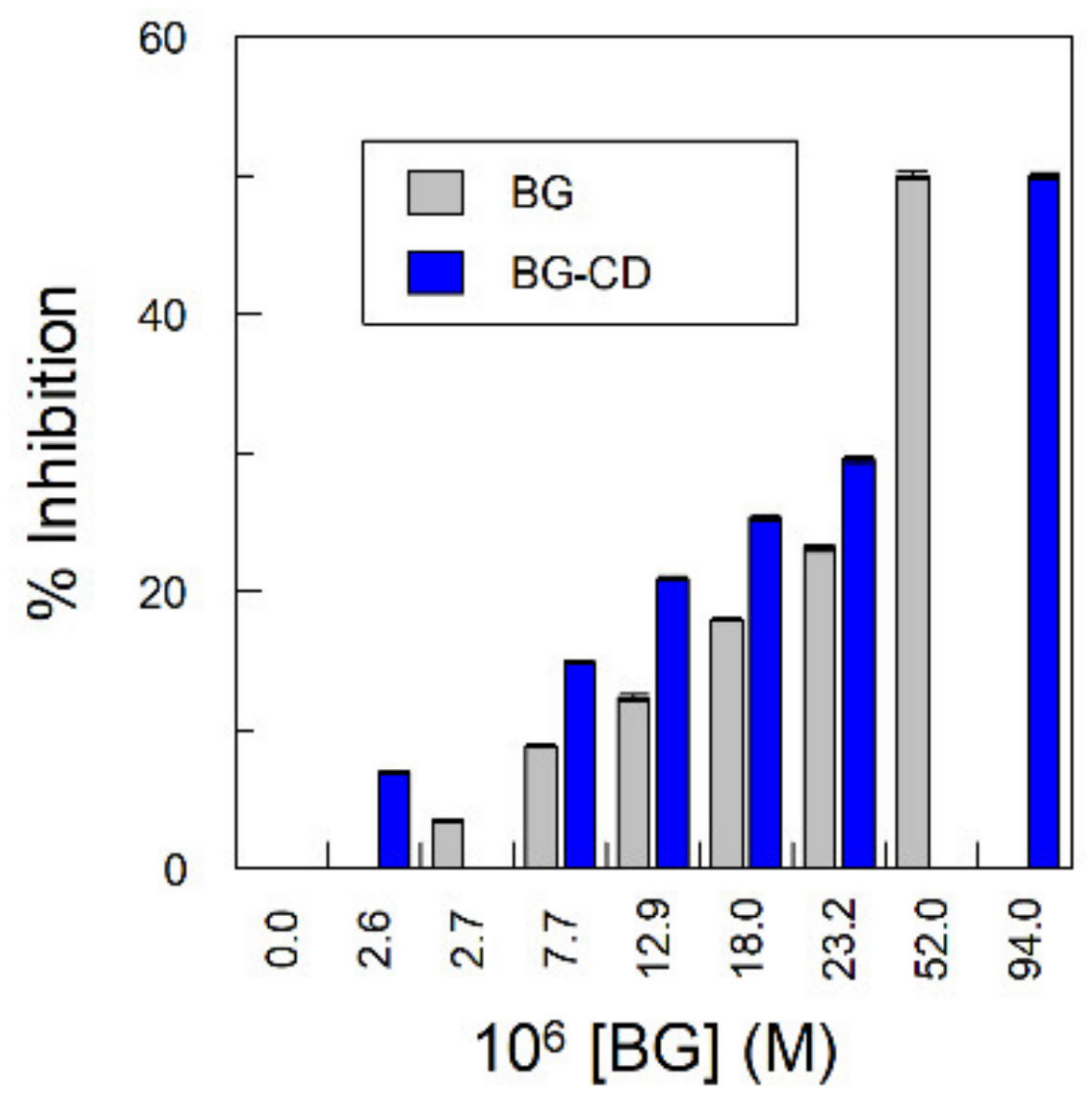
Percentage of inhibition on the 2,2–diphenyl–1pycrilhydrazyl (DPPH^●^) radical content for butyl gallate in the absence (BG) and in the presence (BG–CD) of CDs ([β-CD] = 11 mM), at time 1.8 h and T = 25 ± 1 °C.

Figure 6 is representative and illustrates the effects of [BG] on the percentage of inhibition in the absence and in the presence of β–cyclodextrin. Similar plots were obtained for all gallates (data not shown), and in all cases, %Inhibition values depended on the AO (gallates) and CD concentrations, Figure 6. For instance, %Inhibition increases by a factor of ~4.3 (samples in the presence of CD) or ~6.6 (samples in the absence of CD) on going from [BG] = 2.6 μM to [BG] = 23.2 μM. At a given concentration of gallates, the %Inhibition in the presence of CDs is higher than that in its absence, suggesting that the formation of inclusion complexes with CDs enhances their radical scavenging activity.

The EC_50_ values, defined as the concentration of gallates required to reduce the DPPH^●^ radical concentration by 50% (%Inhibition = 50%) are displayed in Table 2. The higher the EC_50_ value, the lower the antioxidant radical scavenging activity.

Results in Table 2 highlight two relevant points. First, the reactivity of the antioxidants with the DPPH^●^ radical is essentially independent (differences less than 5%) of the length of the alkyl chain but slightly different from that of the parent gallic acid (most likely because of the presence of the carboxylic group, pKa = 4.3). Second, in the absence of CDs, the average value of EC_50_ for the gallates (PG, BG, OG) was 5.5 ± 0.3 M. In the presence of 11 mM β–CD, the EC_50_ values were again independent of the length of the alkyl chain, with an average value of 4.0 ± 0.1 M. Since all gallates form inclusion complexes with β–CD, and given that the EC_50_ values decrease upon addition of β–CD, we can conclude that the radical scavenging activity of complexed antioxidants is higher than those antioxidants that were uncomplexed, i.e., “free”, in the culture medium of BHSCC. These results are in agreement with the observed increase in RSA values of extracellular extracts, Figure 4b, and with literature reports, indicating an increase in the antioxidant activity of encapsulated antioxidants [26,27,28].

## 3. Materials and Methods 

### 3.1. Materials

All chemicals were of the highest purity available and used as received. Gallic acid monohydrate (98.0% pure) and gallates (propyl gallate, PG, butyl gallate, BG, octyl gallate, OG, and lauryl gallate, LG, all 99.0% pure) were purchased from Sigma–Aldrich. 2–Hydroxypropyl–β–cyclodextrin (HP–β–CD) and methyl–β–cyclodextrin (M–β–CD) were purchased form Cyclolab LTD and acetonitrile was received from (ACN, HLPC grade) Merck. The Folin-Ciocalteu’s reagent was obtained by VWR Chemical. Sodium carbonate and 2,2–diphenyl–1–pycrilhydrazyl (DPPH^●^) were purchased from Sigma–Aldrich. Milli-Q grade water (conductivity < 0.1 mS cm^−1^) was employed in the preparation of aqueous solutions. Citric acid and sodium citrate (both from Acros Organics, 99% pure) 0.04 M, pH 3.65, was used as buffer solution.

### 3.2. Plant Material

Epiphyllous buds were harvested in autumn 2018 from 18-month-old *B.* × *houghtonii* plants, cultivated in a local greenhouse (42°12′40.0″ N 8°43′36.1″ W, Vigo, Spain). Buds were maintained in tap water overnight and gently dried with a paper filter at room temperature prior to their surface disinfection, which was carried out inside a laminar flow cabinet under aseptic conditions. The buds were rinsed in 70% (*v*/*v*) ethanol for one minute, washed with sterile distilled water, and then rinsed in 0.4% (*v*/*v*) sodium hypochlorite for 10 min. Finally, buds were washed with sterile distilled water (three times), dried with sterile paper filter to achieve the total removal of surface disinfection agents, as described elsewhere [35]. 

Next, the buds were transferred to culture vessels containing 25 mL of Murashige and Skoog medium (MS medium, Murashige and Skoog basal culture media specifically designed for plant tissue culture) [36], supplemented with 3% (*w*/*v*) sucrose and solidified with 0.8% (*w*/*v*) agar at pH 5.8. Culture medium was autoclaved at T = 121 °C and 1.1 atm for 20 min. Buds were placed in a growth chamber under a photoperiod of 16 h light and 8 h dark, at T = 24 ± 1 °C, and subcultured every 12 weeks into a fresh medium.

### 3.3. Callus Induction

For callus induction, leaf segments (≅1 cm^2^) of in vitro-grown plants (from third subculture) were transferred to plated MS medium with half macronutrient concentration supplemented with 3% (*w*/*v*) sucrose, 0.5 mg/L 2,4–dichlorophenoxyacetic acid and 1 mg/L benzylaminopurine, solidified with 0.8% (*w*/*v*) agar at pH 5.8 (BH medium). Leaf segments were placed in the dark at T = 24 ± 1 °C for four weeks to induce callus formation. Then, calli were spliced from explants and subcultured every 3 weeks under the same conditions.

### 3.4. Establishment of Cell Suspension Culture

For the initiation of BHSCC, 3-week old calli (from fifth subculture) were transferred to 250-mL Erlenmeyer flasks containing 50 mL of liquid BH medium (without agar) at the initial cellular density of 100 g of fresh weight per liter. Cultures were incubated at T = 24 ± 1 °C in the dark in a rotary shaker at 120 rpm and subcultured by half-dilution with fresh medium every 9 days.

### 3.5. Elicitation Experiments and Growth Kinetics Determination

For elicitation experiments, 9-day old BHSCC from third subculture were used. The cellular fraction was separated from culture medium by filtering in vacuum and 2 g of filtered cells were transferred to a 100 mL Erlenmeyer flask containing 20 mL of BH medium (control) or BH medium supplemented with 50 mM methyl–β–CDs (M–β–CD) or 50 mM 2–hydroxypropyl–β–CDs (HP–β–CD). Once initiated, both non-treated and elicited BHSCC were incubated as described above for different periods of times: 0, 2, 4, 7, and 9 days. All treatments were carried out in triplicate. Cells were then separated from the medium by vacuum filtration and both cellular and culture medium fractions were stored at T = −20 °C. Frozen cells were lyophilized, homogenized to get a fine powder, and weighed in order to get growth curves. The average cell growth rate (CGR) was determined as proposed by Pan et al. [37], Equation (2), for a growth time during which the maximum dry cell mass was obtained (Δ*t*).

(2) $$CGR=\frac{{\left(\mathrm{dry}\;\mathrm{cell}\;\mathrm{weight}\right)}_{\mathrm{maximum}}-{\left(\mathrm{dry}\;\mathrm{cell}\;\mathrm{weight}\right)}_{\mathrm{initial}}}{{\left(\mathrm{dry}\;\mathrm{cell}\;\mathrm{weight}\right)}_{\mathrm{initial}}x\Delta t}$$

### 3.6. Phenolic Extraction

In order to perform phenolic extraction of intracellular fraction, 80 mg of lyophilized cells were mixed with 8 mL of 80% (*v*/*v*) MeOH and incubated at T = 60 °C for 10 min. Then, samples were cooled down to room temperature and subjected to sonication for 30 min. Finally, samples were centrifuged at 3500 rpm for 10 min and the supernatant was separated and filtered using PTFE syringe filters (0.22 µm pore size).

For the extraction of extracellular fraction, 4 mL of culture medium were mixed with 4 mL of 80% (*v*/*v*) MeOH and samples followed the same extraction procedure.

### 3.7. Total Phenolic Content (TPC) Determination

Phenolic extracts were analyzed in terms of their phenolic content following the method described by Ainsworth and Gillespie [38]. Briefly, 100 µL of extract were mixed with 200 µL of 10% (*v*/*v*) Folin–Ciocalteu´s reagent for 2 min at room temperature. Then, 800 µL of 0.7 M Na_2_CO_3_ were added and the reaction mixture was vortexed and incubated at T = 25 ± 1 °C for 2 h in the dark. Finally, the absorbance was measured at λ = 765 nm. A calibration curve was performed using GA as standard and results were expressed as total gallic acid equivalents in mg per liter of suspension (GAE, mg/L). All determinations were carried out in triplicate.

### 3.8. Flavonoid Content (FC) Determination

Flavonoid content was analyzed in phenolic extracts following the method described by Pekal and Pyrzynska [39]. Briefly, 1 mL of extract was mixed with 0.3 mL of 5% (*w*/*v*) NaNO_2_ and let stand for 5 min. Next, 0.5 mL of 2% (*w*/*v*) AlCl_3_ were added and, 6 min later, 0.25 mL of 1 N NaOH were added. The reaction mixture was then vortexed and incubated at T = 25 ± 1 °C for 10 min. The absorbance was measured at λ = 510 nm. A calibration curve was performed using (+)-catechin as standard and results were expressed as catechin equivalents in mg per liter of suspension (CE, mg/L). All determinations were carried out in triplicate.

### 3.9. Radical Scavenging Activity (RSA) Determination

The antioxidant activity of gallates and *Bryophyllum* extracts were obtained through the determination of their radical scavenging activity (RSA) using the stable free radical 2.2–diphenyl–1–pycrilhydrazyl (DPPH^●^). DPPH^●^ is a colored free radical which is neutralized under the presence of molecules with antioxidant activity, and consequently, it is bleached in the presence of antioxidants found in plant extracts.

Briefly, the RSA of gallates in the absence and in the presence of CDs was assessed by monitoring their absorbance decrease at λ = 515 nm and T = 25 ± 1 °C. For the purpose, three initial solutions were prepared: a fresh methanolic DPPH^●^ solution 6.2 mM, CD solution 10 mM in a mixture buffered water (citric–citrate 0.04 M pH 3.65)/methanol (9:1, *v*/*v*) and gallates solutions 3.9 mM in the same last aqueous/methanolic mixture. The variation in the absorbance of the DPPH^●^ radical was monitored as a function of time (up to 2 h) for six gallates concentrations ([AO] = 2.6–22.3 μM). An aqueous/methanolic DPPH^●^ solution (9:1, *v*/*v*) with no added antioxidant was used as control. Each set of experiments was performed in triplicate. Auxiliary experiments (not shown) revealed no significant changes in the extinction molar coefficient of DPPH^●^ in the absence and in the presence of CDs (7365 ± 96 and 7904 ± 90 M^−1^ cm^−1^ for DPPH^●^ free and complexed with 12mM β–CD, respectively), indicating that DPPH^●^ molecule was not included in the β–CD cavity.

The RSA of *Bryophyllum* extracts was determined by employing the method developed by Jagtap and co-workers [40]. First, aliquots of 2850 µL of DPPH^●^ methanolic solution 110 µM were added to 150 µL of plant extracts and the mixture was vortexed and incubated at T = 25 ± 1 °C for 24 h in the dark. After incubation, the diminution on DPPH^●^ signal was followed by UV spectroscopy (λ = 517 nm) with the aid of a previously prepared calibration curve.

In both cases (gallates and plant extracts), RSA results were expressed as %Inhibition calculated as Equation (3) where *A*_0_ and *A*_e_ are the absorbance values of DPPH^●^ in the absence and in the presence of polyphenols at a certain time of reaction, respectively.

(3) $$\%Inhibition=\frac{\left(A_{0}-A_{e}\right)}{\left(A_{0}\right)}\times 100$$

### 3.10. Determination of Inclusion Constants (K_inc_) of Gallates with Cyclodextrins (CDs): Spectral Shift and Phase–Solubility Methods

*K_inc_* values for gallates were determined by employing two complementary methods, (a) spectral shift method, based on the changes of absorbance at a given wavelength (λ) upon increasing the concentration of CDs; and (b) phase–solubility method, based on the changes of solubility upon increasing the concentration of CDs. Details of the procedures can be found elsewhere [8].

- Spectral UV–Vis shift method

Spectrums of the gallates ([AO]= 0.1 mM) were obtained in the absence and in the presence of CDs ([CDs] = 0−0.014 M). The increase in the observed absorbance values at fixed λ in the presence of increasing concentrations of CDs was assumed due to the formation of the inclusion complexes. Using Equation (1) and the corresponding mass balances, Equation (4) can be obtained where *A*_0_ and *A* stand for the absorbance for zero and any concentration of CD, respectively, [AO_T_] refers to the stoichiometric AO concentration, and *ε_C_* and *ε_F_* are the molar extinction coefficients for the free and complexed AO form, respectively. According to Equation (4), *K_inc_* (Table 1) and *ε_C_* values were determined from the slope and intercept of these linear plots 1/(A − A_0_) vs. 1/[CD], known as a Benesi–Hildebrand plot.

(4) $$\frac{1}{A-A_{0}}=\frac{1}{\left[AO_{T}](\varepsilon_{C}-\varepsilon_{F}\right)}+\frac{1}{K_{inc}[AO_{T}](\varepsilon_{C}-\varepsilon_{F})}\times \frac{1}{\left[CD\right]}$$

The method cannot be used when there is a low variation in the extinction coefficients of the complexed and free forms of the antioxidants (this is the case, for instance, of gallic acid) or if the guest is very hydrophobic (e.g., octyl gallate) and their solubility in water is very low. In these cases, the inclusion constants (OG–CD) were determined by employing the phase–solubility method described below.

- Phase solubility method

This method was used for determining *K_inc_* values for more hydrophobic gallates. Briefly, volumetric flaks (25 mL) containing 10 mL of an aqueous solutions of increasing [CD] (0–0.02 M) and a specific amount of gallates ([AO] >>> 2 × AO solubility limit in aqueous solution) were placed in an orbital shaker (Incubator Heidolph 1000 with a Heidolph thermostat 1010 to control temperature ± 1 °C, 500 rpm) in the dark and allowed to reach the thermal equilibrium for at least 24 h at T = 25 °C. Bulk solutions were subsequently centrifugated (4000 rpm, 1760× *g*) for 8 min. Here, to determine the concentration of AO in the aqueous phase by UV spectroscopy, a competitive guest molecule method was employed. In this case, acetonitrile (ACN) was used as effective guest competitor with the ability of displace gallates out of the CD central cavity [8]. Aliquots of aqueous phase were diluted in an acetonitrile-buffered water (citric acid–citrate 0.04 M pH 3.65) mixture (8:2, *v*/*v*), their absorbances were measured and the concentration of AO was determined by interpolation in the previously prepared calibration curves in ACN-buffered water mixture (8:2, *v*/*v*).

**Figure 7:**
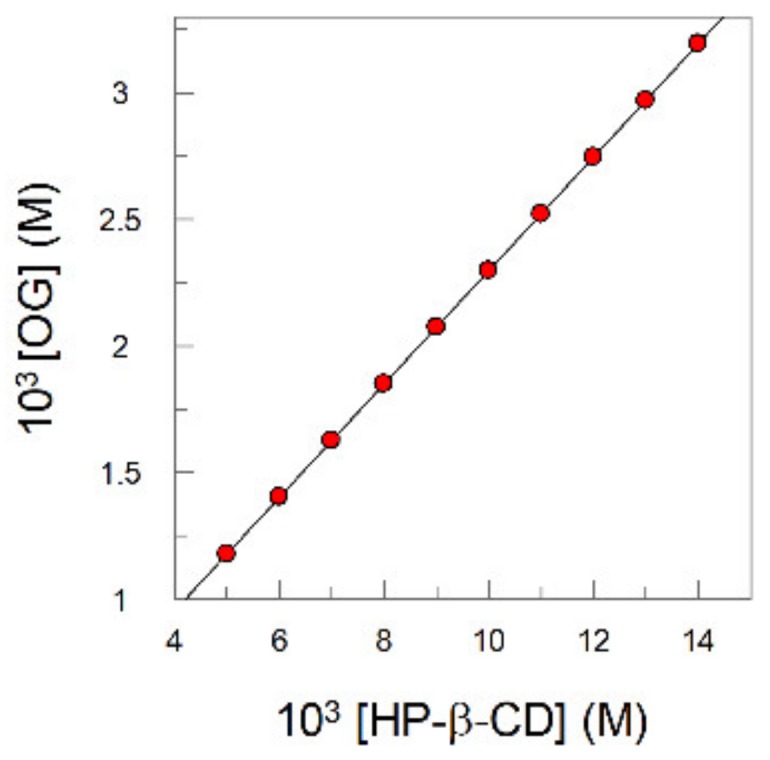
Changes of the concentration of octyl (OG) solubilized in the presence of increasing concentrations of 2–hydroxypropyl–β–cyclodextrin (HP–β–CD) by employing phase-solubility method, T = 25 ± 1 °C.

Assuming that a complex of 1:1 stoichiometry is formed, the concentration of AO is given by Equation (5). Considering the Equation (5) and the corresponding mass balances, Equation (6) can be derived. *K_inc_* values (Table 1) were obtained from the slopes (S) of linear plots of [AO]_measured_ vs. [CD] employing Equation (7). Figure 7 shows a representative plot obtained for OG by employing phase–solubility method. Similar plots were obtained for all gallates. The obtained linear plots corroborate the 1:1 stoichiometry for all gallates, in line with our previous results [8].

Assuming that a complex of 1:1 stoichiometry is formed, the concentration of AO is given by Equation (5). Considering the Equation (5) and the corresponding mass balances, Equation (6) can be derived. *K_inc_* values (Table 1) were obtained from the slopes (S) of linear plots of [AO]_measured_ vs. [CD] employing Equation (7). Figure 7 shows a representative plot obtained for OG by employing phase–solubility method. Similar plots were obtained for all gallates. The obtained linear plots corroborate the 1:1 stoichiometry for all gallates, in line with our previous results [8].


[*AO*]*_M_* = [*AO*]*_F_* + [*AO* − *CD*] = [*AO*]*_F_* + *K_inc_*[*AO*]*_F_*[*AO* − *CD*] (5)

(6) $${\left[AO\right]}_{measured}={\left[AO\right]}_{F}+\frac{K_{inc}{\left[AO\right]}_{F}}{1+K_{inc}{\left[AO\right]}_{F}}[CD]={\left[AO\right]}_{F}+S\left[CD\right],S=\frac{K_{inc}{\left[AO\right]}_{F}}{1+K_{inc}{\left[AO\right]}_{F}}$$

(7) $$K_{inc}=\frac{1}{{\left[AO\right]}_{F}(1-S)}$$

### 3.11. Statistical Analysis

All results are shown as the mean ± standard deviation (SD) of three independent replicates and data were statistically analyzed by independent two-tailed Student’s t test, using STATISTICA v. 12 software (StatSoft, Inc., Street Tulsa, OK, USA, 2014). Significant differences between control and treatments were represented by (*) and were considered when p-value was lower than 0.05 (*p* < 0.05).

## 4. Conclusions

In this work, we applied the PSCC technology for the first time to an unexploited and valuable, with untapped potential, medicinal plant belonging to subgenus *Bryophyllum* (genus *Kalanchoe*). According to our findings, BHSCC elicited with CDs may constitute an efficient system for high-performance applications in Biotechnology. The use of CDs was successfully implemented and has, at least, two main advantages: (1) CDs enhanced, at the same time, BHSCC growth and the biosynthesis of phenolic compounds and (2) CDs formed inclusion complexes with phenolics located in the extracellular medium, shifting the homeostatic equilibrium of the cell, stressing them to produce more phenolics and flavonoids to restore it.

Once released to the extracellular medium, phenolics are complexed by CDs and increase their radical scavenging activity so that the efficiency of this biotechnological system is enhanced in a relatively short time: Only 7 days of culture were sufficient to achieve a stable cell growth and production yields.

The production of phenols and flavonoids in the extracellular medium were ~15–18 times higher when HP–β–CD and M–β–CD were used as elicitors with respect to that in the absence of CDs (control samples). Results also demonstrated that the presence of CDs did not improve significantly the radical scavenging activity of intracellular BHSCC extracts (differences less than 12–17% related to control cells), while that the radical scavenging activity of extracellular BHSCC extracts increases substantially in the presence of CDs.

Overall, our results suggest that the use of elicited suspension cultured cells with CDs may be an optimal starting point to obtain bioactive compounds from *Bryophyllum* extracts under a sustainable and environmentally-friendly procedure, considerably improving *Bryophyllum* potential applications. However, PSCC technology involves a variety of factors that modulate their efficiency and future studies are needed to optimize the use of BHSCC as a robust production system, such as the combination of CD with other elicitors (i.e., jasmonates), the use of different inoculum sizes or the tuning of mineral composition of culture media. As the phenolic compound production is regulated by multiple factors, deciphering the critical ones will be a crucial step for predicting and optimizing phenolic production. In this case, the use of artificial intelligence tools may become a useful tool [41,42]. Further studies with PSCC to analyze these and other factors and to optimize the procedures to satisfy the industry’s demands are in progress and will be part of future reports.

## Figures and Tables

**Table 1:** Inclusion constants, *K_inc_*, for gallic acid and some derivatives and some polyphenols commonly detected in *Bryophyllum* extracts [13]. Values for gallic acid (GA) and its derivatives were obtained in this work by employing (a) the spectral shift method and (b) the solubility method (see Section 3.10). Reported values were calculated assuming a 1:1 stoichiometry in unbuffered water solutions at T = 25 °C, except where otherwise indicated.

Antioxidant (AO)	Cyclodextrin (CD)	*K_inc_* (M^−1^)	AO	CD	*K_inc_* (M^−1^)
Gallic acid	HP–β–CD	551 [29]	Coumaric acid	HP–β–CD	320 [29]
Methyl gallate	HP–β–CD	595 [29]	Quercetin	HP–β–CD	900 [30] (pH 7.4)
Ethyl gallate	HP–β–CD	380 [29]	Catechin	HP–β–CD	8860 ± 270 [31]
Propyl gallate (PG)	β–CD	125 ± 11^(a)^ [8] 105 ± 2 ^(b)^ [8]	Kaempferol	β–CD	580 [32]
	HP–β–CD	343 ± 9 ^(a)^ 328 ± 7 ^(b)^ 250 [29]		M–β–CD	5432 [32]
Butyl gallate (BG)	β–CD	198 ± 13 ^(a)^ [8] 200 ± 15 ^(b)^ [8]		HP–β–CD	6175 [32]
	HP–β–CD	706 ± 38 ^(a)^	Caffeic acid	β–CD	278 [33] (T= 20 °C)
Octyl gallate (OG)	HP–β–CD	4810 ± 98 ^(b)^		HP–β–CD	580 ± 56 [34] (pH 3) 279 ± 11 [34] (pH 6.5)
Ferulic acid	HP–β–CD	555 [29]	Chlorogenic acid	β–CD	424 [33] (T= 20 °C)

Values obtained in this work by ^(a)^ UV shift method, ^(b)^ phase-solubility method (buffered aqueous solution with citric acid-sodium citrate 0.04 M, pH 3.65).

**Table 2:** 2,2–diphenyl–1pycrilhydrazyl (DPPH^●^) radical scavenging activity of gallates tested: gallic acid (GA), propyl gallate (PG), butyl gallate (BG), and octyl gallate (OG) in the absence and in the presence of β–CD (11 mM), T = 25 ± 1 °C.

	10^5^ EC_50_ (M)	
Antioxidants (AOs)	0 M β–CD	11 mM β–CD
**GA**	6.5 ± 0.1	5.8 ± 0.1
**PG**	5.7 ± 0.1	3.9 ± 0.1
**BG**	5.2 ± 0.3	4.1 ± 0.2
**OG**	5.7 ± 0.1	4.1 ± 0.1
